# Domestic Summary

**Published:** 1840-01-25

**Authors:** 


### DOMESTIC SUMMARY.

### *Observations on Dextrine and Diastase.*

By William Procter, jr.

(Continued.)

In conclusion. M. Biot believed that the acid, by the aid of heat, first ruptured the teguments of the globules of fecula, and set the dextrine free, and then, if the heat was continued, acted on the dextrine, and converted it into sugar of amidon, without the acid communicating any of its substances to the product.

In a letter addressed to the French Institute, 1st April, 1838,* M. Guérin announced his having obtained several interesting results with amidon, as follows: that *tegumentary amidon* has the same chemical composition as lignin, and that the *soluble matter* of M. Raspail was composed of two substances, *amidine* and soluble *amidin ;* the first soluble in cold water, and the second insoluble in water, either hot or cold, but capable of being held in solution through the intervention of *amidine.*

MM. Payen and Persoz, in a letter addressed to the Academy, dated 8th of April, that is to say, eight days after the letter of M. Guérin, announced the discovery of *diastase*, the active principle of *malt*, and of the property it possessed of breaking or decomposing the globules of fecula, and setting at liberty the soluble matter, or dextrine. "This substance," (diastase,) they say in their letter, "contains the less azote as it approaches a state of purity, and possesses besides the following properties: it is solid, white, insoluble in alcohol, soluble in water ; its solution is neuter, and its taste marked : it is not affected by the subacetate of lead : abandoned to itself for some time, it becomes acid ; heated to 150° or 160° Fahr., with fecula, it possesses the *remarkable power* of breaking, instantly, the envelopes, and setting at liberty the dextrine, which dissolves easily in water, whilst the teguments, insoluble in this liquid, float or precipitate according to the density of the liquor. This operation, properly managed, gives *pure dextrine*, which possesses a great power of rotation, unequalled by that obtained by any other process. When diastase is present in a solution of dextrine, it always converts the latter substances into sugar, provided the temperature is not elevated during their contact above 160° or 170° Fahr., because, if heated to ebullition, it loses the property of acting on fecula and dextrine."

"Diastase exists in the germinated seeds of

* Annal. de Chem. et de Phys. tome ix, 1834.

barley and wheat, and in the germs or *eyes* of the potatoe, (*Solanum tuberosum*,) where it is always accompanied by an azotized substance which is soluble in water, insoluble in alcohol, capable of being coagulated by heat, incapable of acting on fecula, and of being precipitated from its solution by subacetate of lead."

The following is the process for preparing diastase announced by Payen and Persoz in the same communication :—One part of malt (*d'orge germée*) is reduced to powder, and mixed in two and a half parts of distilled water. After macerating for a few minutes, the mixture must be thrown on a filter. The liquid thus obtained is to be heated to 150° Fahr., when the azotized substance is coagulated, and can be separated by filtration. The liquid then contains diastase and sugar, in quantity in proportion to the action of the diastase on the dextrine of the malt in the germinating process. It is then precipitated by alcohol, which holds the sugar in solution. This precipitate should be redissolved and again precipitated to obtain it pure.

In a subsequent communication,* Payen and Persoz state that the soluble matter obtained according to their process is not a pure substance, but is constituted of dextrine, (properly speaking,) sugar, and a substance analogous to inulin.

In a yet more recent communication† Payen and Persoz declare that fecula is composed of *amidone* and *teguments*, and hence they say amidone is a natural product, and can be obtained by means of water alone.

*Amidone* is insipid, neuter, without colour, diaphanous, elastic, and tenacious. It is insoluble in cold water, but swells exceedingly, and appears in solution; and in this state forms a blue colour with iodine, is precipitated by tannin, infusion of galls, subacetate of lead, lime, and baryta. The barytic compound is soluble in an excess of either hot or cold water, and by precipitating the baryta with a reagent, the amidone can be obtained in so minute a state of division that its combination with iodine is not precipitated by saline solutions.

It can be obtained by boiling one part of fecula in 100 parts of water, filtering through a double filter, and evaporing carefully to dryness; redissolving, filtering, and again evaporating to dryness; the product is amidone.

Diastase, in its action on fecula, converts the amidone into gum, or dextrine and sugar. The dextrine and sugar obtained by diastase, are not precipitated by tannin, subacetate of lead, lime, baryta, or infusion of galls. The sugar can be separated from the dextrine by alcohol of .84. It has a very sweet taste, and ferments with yeast, yielding alcohol and carbonic acid, which is not the case with dextrine. It is dextrine which communicates to beer the mucilaginous property which holds the carbonic acid, and which gives to the froth its persistence.

The teguments contain carbonate and phosphate of lime and silica.

The jelly (*empois of the French*) yielded when fecula is boiled in water, is accounted for by the swelling of the amidone when in contact with the water, after the rupture of the envelopes. When diastase is added to this gelatinous mass, it has the singular property of destroying its spongy organic character, and of rendering it perfectly soluble by converting it into dextrine and sugar.

In conclusion, MM. Payen and Persoz remark,—

1st. That the fecula of the grains and potatoes are formed of *amidone* and *teguments*.

2d. That the teguments vary in different feculæ, by the presence and proportion of an acrid, disagreeable, tenacious substance, which communicates to them their special taste.

3d. That in the action of water, lime water, iodine, baryta, and diastase, these substances pass through the envelope.

4th. That amidone is chemically identical in different feculæ, but varies in its volume and cohesion.

5th. That it is *insoluble in the cold*, but allows water to penetrate between its particles, and swell them by degrees.

6th. That it is spongy in its texture, and when considerably broken up by strong ebullition, in 100 times its weight of water, it appears to be really dissolved, and passes the filter.

7th. That the previous observations explain the formation of jelly, and its different characters when obtained from different feculæ.

8th. That the teguments, entirely deprived of amidone, do not yield a blue colour with iodine.

9th. That amidone *alone*, in fecula, produces alternately, the phenomena of colouration, and decolouration, of opacity and diaphaneity, by iodine, alcohol and water.

10th. That amidone *only* of fecula, is transformed into sugar, and gum, by the influence of diastase, water, and temperature, and that the sum of the weights of the sugar, and gum, or dextrine, equals the weight of the amidone.

11th. That in the preparation of beer, it is important to effect the entire transformation of the amidone, into dextrine and sugar; so that the transparency of the fluid will not be troubled afterwards, which would be the case if amidone was present.

12th. That in the first development of certain plants, the diastase is placed precisely at the point where the fecula ought to be rendered assimilable so as to transform the insoluble fecula into two soluble substances, capable of being absorbed by the plant.

* Journal de Pharmacie, Mai, 1834, (note by Soubeiran.)

† Annales de Chemie et de Physique, tome lvi, Aout, 1834.

13th. That diastase only transforms amidone, as it is without action on gum, (properly so called,) inulin, albumen, gluten, teguments, and lignin.

14th. That during the process of germination, the proportion of the diastase increases with the development of the gemmule.

15th. That amidone forms about .995 of fecula, while the teguments, deprived of all amidone, make up the remainder.

Finally, that diastase has become an important agent in the chemical analysis of fecula, and forms an easy means of making commercial dextrine.

M. Guérin says, that he followed M. Chevreul in naming the principles which he has described,* that is to say, *amidine* to the portion of fecula soluble in cold water; *tegumentary amidin* to the part insoluble in either cold, or boiling water; and *soluble amidin*, to prevent all confusion, to the part which is held in solution by amidine, and which is identical in chemical composition with tegumentary amidin.

The amidine of Guérin, is made by boiling fecula in a large quantity of water for a short time, allowing it to stand until the teguments precipitate, filtering, and evaporating the filtrated liquor to a syrupy consistence, when a precipitate gradually forms, which is to be separated by a cloth, and the liquid again evaporated, continuing the process until it ceases to yield the insoluble matter. When finally evaporated to dryness, the residue is completely soluble in cold water. M. Biot found a solution of this substance to possess the rotatory power three times more powerfully than sugar.

It renders cold water very mucilaginous, is insoluble in alcohol and sulphuric ether; its aqueous solution becomes acid, after standing many days, and its transparence is troubled.

Treated with nitric acid, it gives first oxalhydric, and afterwards oxalic acid.

100 parts of amidine, and 250 parts of sulphuric acid, at about 150° Fahr. have yielded 95.8 parts of anhydrous sugar.

M. Guérin says that this substance differs much from the dextrine of Biot and Persoz, because, according to their memoir, they assign as one of its chemical characters, fermentation with yeast, which amidine does not. He prepared some of their dextrine, and found that it did ferment with yeast; but suspecting that it owed this property to sugar, he treated it with alcohol and found that it lost this property, and acquired that of colouring blue with iodine, instead of vinous red or purple. Thus deprived of sugar by alcohol, continues Guérin, it is yet composed of two substances, one soluble and the other insoluble in cold water. Biot and Persoz say, that their dextrine is the same as that made with boiling water alone, which corresponds with the declaration before mentioned of Payen and Persoz, that the substance obtained by diastase is composed of dextrine, sugar, and substance analogous to inulin.

*Tegumentary amidin*, of Guérin, dried at 212° Fahr. has a light yellow colour neither odour nor taste, without action on paper reactives, gives a beautiful blue colour with iodine, is insoluble in water, either cold or boiling, alcohol, or sulphuric ether, and when left long in contact with water, swells, but is not altered.

*Soluble amidin*, of Guérin, is the substance precipitated from the aqueous solution of amidon, in the process for preparing amidine; and according to that chemist, it is chemically identical with tegumentary amidin. He also observes that fecula is composed of—

| | | |
|---|---|---|
| Tegumentary amidin, | 2.2 | 100.00 |
| Soluble amidin, | 38.68 | |
| Amidine, | 59.12 | |

Guérin also queries whether his tegumentary amidin is identical with lignin, and owes its property of colouring blue with iodine to a small quantity of adhering amidine, or whether it is merely isomeric with that substance.

We have now gone over most of the writers on the subject before us, and have unfolded their several views as concisely as possible. It now remains to give the ideas of M. Raspail, the latest writer on the subject whose work has come to hand. He differs widely in his opinions from most of those which we have detailed, and it is proper that they also should be exposed: avoiding, however, the many sarcastic remarks with which his pages are interspersed, we shall endeavour to place his views in as clear a light as possible.

According to this writer, fecula consists of soluble matter and teguments; the latter enveloping the former; the one entirely insoluble in water, and the latter very soluble in that fluid. To the soluble matter he gave the name of *soluble substance of fecula*; and to the insoluble portion, *teguments*; stating, at the same time, that the latter consisted of the outer envelope, and an interior tissue, which, from its extreme tenuity, was capable of being diffused through the solution of the soluble substance, so as to appear in solution, though it really was not, and in time was wholly deposited.

He queries what is the *dextrine* of Biot?* and answers that it is the *soluble substance* of fecula.

He again queries what is the *diastase* of Payen and Persoz? after speaking of the unhappy application of this word to the substance in question, which signifies division, or separation, and is already used in chirurgy to signify *luxation*, he remarks, that the diastase of these chemists is distinguished by two properties new in science, and extremely remarkable: the first is its power of breaking the integuments;

* Annales de Chim. et de Physique, tome lvi.

* Nouveau Systeme de Chimie Organique, tome i, art. Amidon.

the second is that of saccharifying fecula. Now unhappily the first property is possessed by pure water, which, when heated to a certain degree, and fecula is thrown into it in small quantities, the envelopes are burst, and the soluble substance is liberated. After standing, the teguments subside.

"The second property," continues Raspail, "which consists in saccharifying fecula, is highly interesting, and is incontestible, but unfortunately it is nothing new. It belongs in justice and without any modification, under an economical relation, to those who have invented and perfected the art of fabricating beer; and under a scientific relation, to Kirchoff, who demonstrated by the most varied experiments, the influence that, not only glutinous substances, but solution of malted barley, that is to say, germinated barley, exercised on the saccharification of fecula."

Payen and Persoz say, that their diastase is not gluten, because it is soluble in water, which is not the case with that substance.

Raspail then observes that "gluten is soluble to a certain degree in water saturated with an acid, or ammonia: now in organized nature so abundant in acid, and ammoniacal products, gluten ought often to be presented under this soluble form, and it has not escaped anterior observations. Thus, before the name of *diastase*, it had received the name of *soluble gluten*, properly so called by Einhoff and Berzelius, that of *zimome* by Taddeï, and that of *legumine* by Braconnot.

Raspail then remarks that in 1826 and 7 he demonstrated that grain, in germinating, produces an energetic acid, which is the acetic, and that then the gluten loses its consistence, and the feculent perisperm becomes milky, which is affected gradually by the acetic solution of gluten, and the teguments are emptied of the soluble substance. The results of the action of gluten on fecula, is to transform it into sugar, then into alcohol, and lastly into acetic acid. The brewers arrest the fermentation at the first two phases. They cause the barley to germinate until the plumule has attained a little greater length than the grain, then dry it and reduce it to powder, and then dissolve it in warm water. The meal thus obtained is *malt*, and the solution, which is the saccharifying agent of the brewers, is the *diastase*, the *zimome*, and the *legumine* of others. The diastase, therefore, of Payen and Persoz, is only a mixture, more or less variable and complex, of sugar, gum, soluble substance of fecula, oil, salts, and finally gluten, to which belongs specially the saccharifying power. Again our author queries:

"What is the *amidine* of Guérin?"

"It is the *soluble substance* that we have discovered in fecula, that we have designated under the name *gummy substance*, to which Biot has believed it his duty to give the name of *dextrine*, a name which Payen and Persoz have added to the soluble substance altered by the malt of beer, which they have since abandoned for that of amidone." This is incorrect, as they do not use diastase in the preparation of amidone.

"What is the *tegumentary amidin* of Guérin?"

"It is the assemblage of the envelopes of fecula, that we have designated under the name of *teguments*."

"What is the *soluble amidin* of Guérin?"

"It is the same, according to him, as tegumentary amidin, which amidine holds in solution, and which according to the same author is identical in all its relations with tegumentary amidin; thus we have two substances which are identical, and different, and one substance which changes its name according as it is dissolved or undissolved."

"It is the interior tissue spoken of before, which from its extreme tenuity passes the filter, and appears dissolved, but which precipitates by time, that Guérin has given the name of soluble amidin."

Perhaps it will give a better idea of these substances by presenting them in the following classification, viz.:

| | | | |
|---|---|---|---|
| Raspail, | teguments, | interior tissue, | soluble substance or gummy substance. |
| Biot and Persoz, | teguments, | substance analogous to inulin, | dextrine. |
| Guérin, | tegumentary amidin, | soluble amidin, | amidine, |
| Payen and Persoz, | teguments, | amidone: substance analogous to inulin. | amidone: dextrine or gum. |

(*To be continued.*)

